# Recombinant Baculovirus: A Flexible Drug Screening Platform for Chikungunya Virus

**DOI:** 10.3390/ijms22157891

**Published:** 2021-07-23

**Authors:** Muhammed Muhsin Varikkodan, Chun-Chung Chen, Tzong-Yuan Wu

**Affiliations:** 1 Department of Chemistry, Chung Yuan Christian University, Chungli 320, Taiwan; muhammedmuhsinv@gmail.com; 2 Department of Bioscience Technology, Chung Yuan Christian University, Chungli 320, Taiwan; chrismouse910093@hotmail.com

**Keywords:** chikungunya virus, baculovirus, BacMam system, membrane fusion, drug screening, antiviral drugs, baicalin, baicalein

## Abstract

Chikungunya virus (CHIKV) is a mosquito-transmitted infectious agent that causes an endemic or epidemic outbreak(s) of Chikungunya fever that is reported in almost all countries. This virus is an intense global threat, due to its high rate of contagion and the lack of effective remedies. In this study, we developed two baculovirus expression vector system (BEVS)-based approaches for the screening of anti-CHIKV drugs in *Spodoptera frugiperda* insect (Sf21) cells and U-2OS cells. First, structural protein of CHIKV was co-expressed through BEVS and thereby induced cell fusion in Sf21 cells. We used an internal ribosome entry site (IRES) to co-express the green fluorescent protein (EGFP) for identifying these fusion events. The EGFP-positive Sf21 cells fused with each other and with uninfected cells to form syncytia. We identified that ursolic acid has potential anti-CHIKV activity in vitro, by using this approach. Second, BacMam virus-based gene delivery has been successfully applied for the transient expression of non-structural proteins with a subgenomic promoter-EGFP (SP-EGFP) cassette in U-2OS cells to act as an in vitro CHIKV replicon system. Our BacMam-based screening system has identified that the potential effects of baicalin and baicalein phytocompounds can inhibit the replicon activity of CHIKV in U-2OS cells. In conclusion, our results suggested that BEVS can be a potential tool for screening drugs against CHIKV.

## 1. Introduction

Chikungunya fever is a viral, mosquito-borne disease caused by an alphavirus from the *Togaviridae* family. The enveloped, positive strand RNA virus is transmitted to humans mainly by *Aedes albopictus* and *Aedes aegypti* mosquito species [1]. It has been seen as an epidemic threat over the past fifteen years (since 2004), engendering some mortalities and associated with severe and chronic morbidity [2,3]. The major chikungunya virus (CHIKV) genotypes have been classified as Asian, West African, and East Central South African are based on their geographical distributions [4]. The large amount of CHIKV outbreaks occurred in the different parts of Africa, south-east Asia (India, Indonesia, Singapore and Philippines) and few are even reported from European countries [5,6]. In 2005–2006, one third of La Reunion Island residents from France, and in 2013, a Caribbean population were infected by an outbreak of chikungunya disease, which has become a common health threat in Central America and Western Europe [7,8]. These two outbreaks caused hundreds of deaths and more than a million people were ill [9]. CHIKV infection causes a feverish illness like Dengue virus and leads to symptoms such as high fever, muscle pain, joint pain, headache, nausea, fatigue, vomiting, conjunctivitis and rashes, and is rarely fatal in humans [10,11]. The United States armed force has considered that CHIKV may be a biological weapon due to its biosafety level 3 (BSL3) pathogenicity. In addition, the National Institute of Allergy and Infectious Disease (NIAID) in the United States has designated it a priority pathogen in category C [12].

The linear, positive-sense, single-stranded RNA genome of CHIKV has around a 11.8-kb size [13,14]. CHIKV RNA contains both 49 S genomic RNA and 26 S subgenomic RNA [15]. It consists of the coding of two open reading frames (ORF) for four nonstructural proteins (nsP1 to nsP4), three structural proteins (capsid, E1 and E2) and two minor cleavage products (E3 and 6K) [16,17]. The nonstructural proteins nsPs 1, 2, 3 precursor (nsP123) and nsP4 function in a complex for viral negative-strand RNA synthesis, after which the sequential processing of nsP123 into its individual proteins results in positive-strand RNA transcription and the production of subgenomic RNA [18]. The structural polyprotein is translated from this subgenomic RNA and contains capsid and envelope glycoproteins (E1 and E2) that constitute the virus particle [18,19]. The glycoproteins are arranged in 80 trimeric spikes and each spike consists of three E2/E1 heterodimers [20]. Trimeric spikes are essential for the budding of new virus particles, host receptor recognition and attachment (via E2), and cell entry via pH-dependent endocytosis (via E1) [21]. The E1 envelope protein is a class II fusion protein that mediates low pH-triggered membrane fusion during virus infection. The E2 envelope proteins are type I transmembrane glycoproteins responsible for receptor binding [20,22]. The capsid protein C is autocatalytically cleaved from the structural polyprotein and encapsulates cytoplasmic viral genomic RNA. The envelope polyproteins (E3, E2, 6K, and E1) are processed in the endoplasmic reticulum (ER) of the cell [21,23]. Researchers have proposed that alphavirus E3 is involved in the processing of envelope glycoprotein maturation, whereas alphavirus 6K has been implicated in envelope protein processing, membrane permeabilization, virion assembly, and virus budding [24,25]. The sequence-specific manner interaction of both 6K and PE2 or E1 leads to forming an efficient virus budding [26]. The 6K allows lipids from the membrane to flip from one side of the bilayer to the other during virus budding. Mostly, 6K is excluded from integration into new virions; however, mutations in 6K leads to decreasing virion production with impaired fusion activity and core deformations [27,28]. CHIKV can also be spread through cell-to-cell transmission, which allows virions to efficiently avoid attacks from the immune system of the host [29].

Baculovirus is an enveloped, double-stranded DNA virus belonging to the Baculoviridae family. This baculoviridae family is gnomically diverse baculovirues, with a genome ranging from about 90 kbps to 180 kbps, and has been reported to infect more than 500 insect species [30]. This baculovirus-insect cells based recombinant DNA technology has proved to be a valuable vector for the production of recombinant proteins [31]. Until now, thousands of recombinant proteins, including cytosolic enzymes and membrane-bound proteins, have been successfully produced through a baculovirus expression vector system (BEVS) [32]. To explore the antiviral activities of wards of chemicals, as well as plant-derived compounds they were studied via BEVS and in particular the anti-CHIKV activities of suramin, niclosamide and nitazoxanide were proved by adopting this approach [19,33,34,35]. Antiviral drugs have the ability of disrupting the alphavirus life cycle. These drugs can mainly alter four steps of viral multiplication (binding, fusion, genome synthesis and transmission (as well as release and cell-to-cell transmission)) [34,36,37]. The infection of an alphavirus is established via receptor-mediated endocytosis. Virus membrane fusion is an essential step of CHIKV infection for viral genome release into the host cell [38]. CHIKV fusion and the budding process are dependent on lower levels of pH and the presence of cholesterol—as is the case for Semliki forest virus (SFV) and Sindbis virus (SINV) [19,34,35,39,40]. The membrane fusion events on CHIKV infection are mediated by class II fusion proteins, specifically involving structural E1 glycoproteins [41,42]. The lower level of pH in the environment of the endosome leads to the conformational changes of envelope proteins, E2–E1 heterodimers dissociation and E1 glycoprotein trimerization that occur during membrane fusion [43,44]. Crystal structures of CHIKV and SINV envelope proteins have also been reported [23,45]. The extended intermediate of the E1 protein inserts into the target membrane through its hydrophobic fusion peptide, and is then trimerized and refolds to form a hairpin-like structure that leads to the fusion process [34,46].

In the present study, we exploited the use of a CHIKV 26S-mediated insect cell fusion inhibition assay through a baculovirus-based expression vector system as a drug screening platform to search for novel anti-CHIKV drugs. We also used the BacMam virus system to co-express CHIKV non-structural proteins (nsPs) and EGFP (enhanced green fluorescence protein) to identify EGFP-positive mammalian cells that can simultaneously express nsPs on their cell surfaces. We identified the effects of three potential anti-CHIKV candidates, ursolic acid, baicalin, and baicalein. This work suggested that the antiviral properties of these plant-derived compounds made them favorable compounds for the development of anti-CHIKV drugs. In addition, our results confirmed cell fusion induced by baculovirus-based expression of structural proteins and that inhibition of CHIKV replication by BacMam virus-based non-structural proteins can be used to screen for CHIKV inhibitors.

## 2. Results

### 2.1. Construction of Recombinant Baculovirus Vectors Can Express CHIKV Structural and Non-Structural Proteins

**Figure 1:**
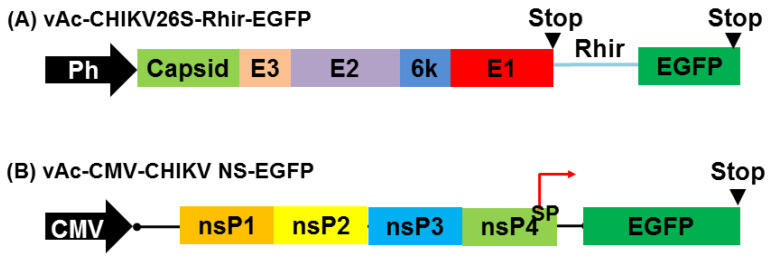
Schematic presentation of the recombinant baculovirus transfer vector and BacMam vector used in this study. (**A**) vAc-CHIKV26S-Rhir-EGFP is a baculovirus that expresses CHIKV structural proteins controlled by Ph promoter. (**B**) vAc-CMV-CHIKV NS-EGFP BacMam vector contain human cytomegalovirus immediate early promoter (CMV) to control the expression of CHIKV non-structural protein and the reporter gene EGFP controlled by subgenomic promoter. (Abbreviations: Ph, polyhedrin promoter; EGFP, enhanced green fluorescent protein gene; Rhir, RhPV 5′-UTR IRES; STOP, translational stop codon, CHIKV structural proteins (Capsid, E3, E2, 6k and E1); CHIKV non-structural proteins (nsP1–4); and SP, subgenomic promoter).

It is pertinent to develop drugs against CHIKV for its control and prevention. The baculovirus expression vector system has proved to be a valuable technology for the production of recombinant proteins. Here, a bi-cistronic recombinant baculovirus, vAc-CHIKV26S-Rhir-EGFP (Figure 1A), was established and used to co-express CHIKV structural proteins with EGFP in *Spodoptera frugiperda* insect cells (Sf21). vAc-CHIKV26S-Rhir-EGFP contains the full-length cDNA of CHIKV 26S subgenomic RNA encoding all the structural proteins, including Capsid, E3, E2, 6k and E1. It was expressed and processed well in vAc-CHIKV26S-Rhir-EGFP-infected Sf21 cells, as mentioned before [19,35]. The co-expression of EGFP with CHIKV structural proteins from bi-cistronic baculovirus expression vector not only simplified the purification of recombinant baculoviruses vAc-CHIKV26S-Rhir-EGFP, but also made detection of the infected cells easier. EGFP-positive cells facilitate the characterization of syncytia. Furthermore, we have evidenced that vAc-CHIKV26S-Rhir-EGFP-infected Sf21 cells can induce cellular fusion and determined that suramin, niclosamide, and nitazoxanide possessed anti-CHIKV abilities [33,34]. All the compounds that we identified through the inhibition of cellular fusion infected by vAc-CHIKV26S-Rhir-EGFP in Sf21 cells are repurposing drugs. However, this method does not yet test natural compounds. In addition, to explore other routes that can block the replication of the CHIKV, vAc-CMV-CHIKV NS-EGFP containing CHIKV non-structural proteins (nsP1–4) and EGFP gene flanking the subgenomic promoter (SP) under the control of cytomegalovirus (CMV) promoter, a vector system was constructed, as shown in Figure 1B. We proposed that vAc-CMV-CHIKV NS-EGFP-transduced mammalian cells like U-2OS can express the replicon of CHIKV and drive the expression of EGFP through SP promoter. These vectors are used to screen the potential effects of natural compounds that can block the replication activity of the CHIKV replicon through the EGFP expression system. When we screened 17 natural compounds (Supplementary Materials: Table S1) for their potential to be used to prevent CHIKV infection, only ursolic acid inhibited the cell-fusion event in vAc-CHIKV26S-Rhir-EGFP-infected Sf21 cells, and baicalin and baicalein inhibited replicon activity in vAc-CMV-CHIKV NS-EGFP-transduced U-2OS cells (Supplementary Materials: Table S1).

### 2.2. Establishment of CHIKV 26S-Mediated Insect Cell Fusion Inhibition Assay

**Figure 2:**
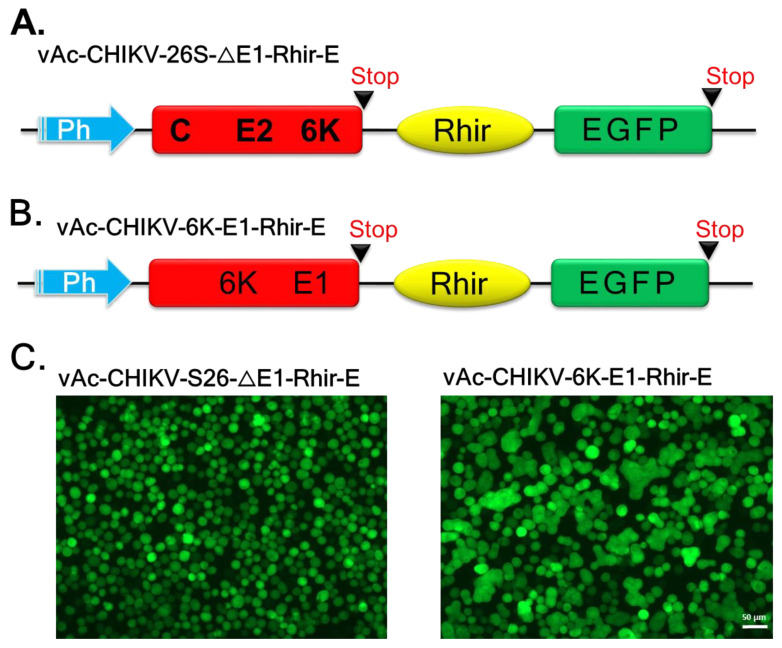
The E1, but not E2, of CHIKV mediated the cell fusion events in insect Sf21 cells. (**A**) vAc-CHIKV-S26-ΔE1-Rhir-E, the recombinant baculovirus carrying the truncated CHIKV 26S in which the E1 carboxyl terminal with 89 amino acids were deleted. (**B**) vAc-CHIKV-6K-E1-Rhir-E, in which the recombinant baculovirus carrying 6K-E1 gene cDNA (a 1.8-kb DNA fragment. (**C**) Sf21 cells (2 × 10^5^ cells per well seeded in a 24-well plate) were infected with the indicated viruses at a MOI of 5 and observed at 3 dpi under fluorescence microscopy. Cell fusion was observed under a fluorescence microscope with an FITC channel (green). Both pictures were taken at an exposure time of 260 ms. Bar represents 50 um. Ph, polyhedrin promoter; EGFP, enhanced green fluorescent protein gene; Rhir, RhPV 5′ UTR IRES.

**Figure 3:**
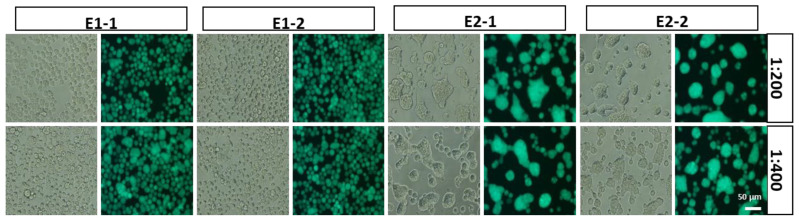
The effects of mAbs against E1 of CHIKV (E1-1 and E1-2, **left**) and against E2 of CHIKV (E2-1 and E2-2, **right**) on cell fusion inhibition. Sf21 cells were infected with vACMVpH-CHIKV26S-Lir-EGFP at MOI of 1, expressed in Sf21 cells analyzed by the indicated monoclonal antibodies in 200-fold and 400-fold dilutions, respectively. vACMVpH-CHIKV26S-Lir-EGFP-infected Sf21cells were examined under a fluorescence microscope with a FITC channel (lower panel) and bright field (upper panel). The bar represents 50 µm.

**Figure 4:**
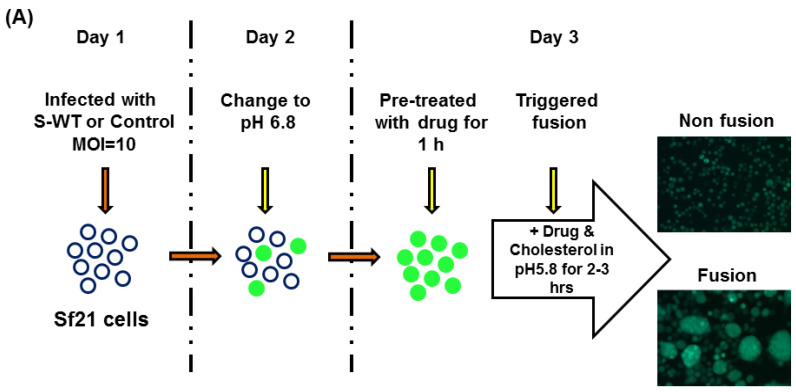

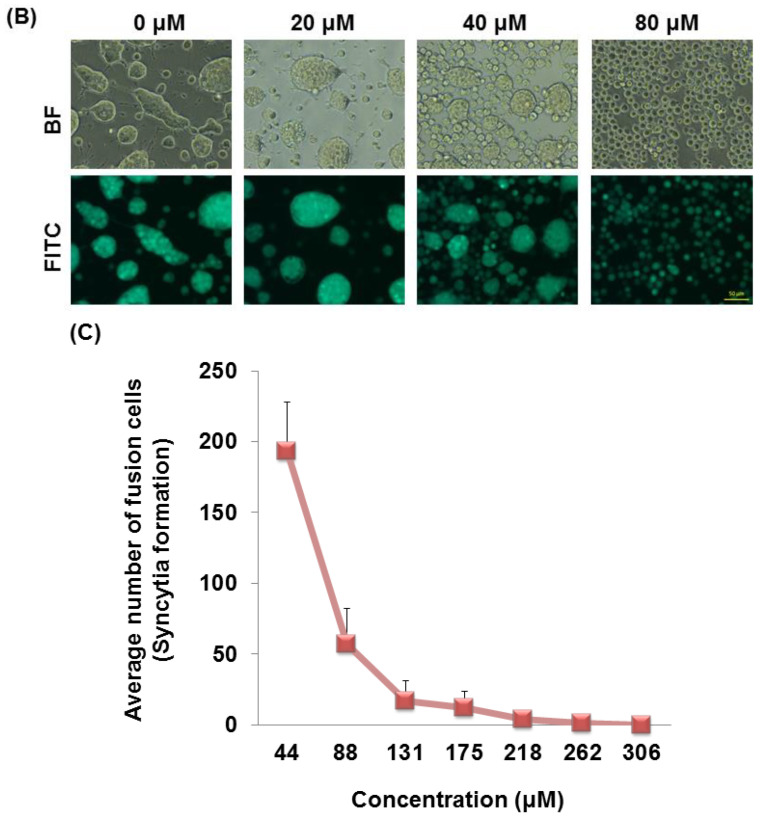
Establishment and analysis of recombinant baculovirus-mediated insect cell fusion inhibition assay. (**A**) Schematic representation of the CHIKV 26S-mediated insect cell fusion inhibition assay. (**B**) Baculovirus vector vAc-CHIKV26S-Rhir-EGFP infected to Sf21 cells at pH 5.8. The effect of ursolic acid on CHIKV 26S-mediated Sf21 cell fusion in 0, 20, 40, 80 µM concentrations were examined under a fluorescence microscope with a FITC channel (lower panel) and bright field (upper panel). Pictures from upper and lower panels were taken in the same field. Sf21 cells were infected with recombinant baculovirus of vAc-CHIKV26S-Rhir-EGFP, at multiplicity of infection MOI of 10. The bar represents 50 µm. (**C**) Dose–response curve of ursolic acid on CHIKV 26S mediated Sf21 cell fusion. Dose dependent curve shows anti-CHIKV effects of Ursolic acid. The fusion was calculated by counting the number of each cell–cell aggregation contain at least three nuclei as syncytia formations and normalizing the value with respect to that of the virus control group (mock), all the data were replicated 3 times.

An illustration of CHIKV 26S-mediated insect cell fusion inhibition assay protocol is shown in Figure 4A. The EGFP-positive Sf21 cells fused with each other and with uninfected cells to form syncytium, and was supported by cholesterol and a lower level of pH (5.8) [34]. The fusion phenomenon was specifically triggered by CHIKV structural proteins, such as E1 and E2, in the presence of cholesterol at lower pH levels [20,34]. To clarify the role of E1 and E2, vAc-CHIKV-26S-△E1-Rhir-E and vAc-CHIKV-6K-E1-Rhir-E were constructed, as describe before [19]. vAc-CHIKV-26S-△E1-Rhir-E was the recombinant baculovirus that expressed all structural proteins of CHIKV except the E1 protein (Figure 2A). In contrast, vAc-CHIKV-6K-E1-Rhir-E was the recombinant baculovirus that only expressed the E1 6K proteins (Figure 2B). Figure 2C shows that as the cell fusion events disappeared in the vAc-CHIKV-26S-△E1-Rhir-E-infected Sf21 cells (left panel), the cell fusion events were maintained in the vAc-CHIKV-6K-E1-Rhir-E-infected Sf21 cells. Thus, the E1 protein is essential and sufficient for the cell fusion observed in vAc-CHIKV26S-Rhir-EGFP-infected Sf21 cells [19,35], see Figure 4. Furthermore, we used the vAc-CHIKV26S-Rhir-EGFP-infected Sf21 cells to analyze monoclonal antibodies (mAbs, E1-1 and E1-2 against E1 protein of CHIKV; E2-1 and E2-2 against E2 protein of CHIKV, the antibodies were a kind gift from Dr. Pei-Yun Shu, Center for Diagnostics and Vaccine Development, Centers for Disease Control, Ministry of Health and Welfare, Taiwan) against the E1 and E2 proteins of CHIKV, respectively. Interesting, we found that the mAbs against the E1 proteins inhibited fusion events; however, the mAbs against the E2 proteins did not interfere in cell fusion (Figure 3). Thus, these results implied that the candidate compound(s) that will block the cell fusion event in vAc-CHIKV26S-Rhir-EGFP infected Sf21 cells may work like the anti-E1 antibodies that inhibit cell fusion.

First, we investigated whether this novel BEVS can act as a screening tool for natural compounds that have potential use for the treatment of Chikungunya fever. The BEVS screening system was previously employed for the identification of the antiviral activities of chemical libraries [34,47]. However, to the best of our knowledge, there are no reports on natural compounds or plant-derived compounds regarding anti-CHIKV effects adopting the BEVS system. We identified that ursolic acid (UA), a plant-derived compound, can inhibit cellular cell–cell fusion events by recombinant baculovirus vAc-CHIKV26S-Rhir-EGFP-infected Sf21 cells. To assess the anti-CHIKV activity of UA, we used vAc-CHIKV26S-Rhir-EGFPinfected insect cell to conduct a fusion inhibition assay. We used different concentrations of UA (20, 40, and 80 μM) on virus fusion triggered at pH 5.8, MOI 10 (Figure 4B). The UA treatment showed that the cellular fusion event was inhibited by UA and all the fusion events disappeared at a concentration of 80 µM. Further, for the dose-related assay, recombinant baculovirus-infected Sf21 at MOI 10 were treated with 44, 88, 131, 175, 218, 262 and 306 μM doses of UA at a 2–3 h incubation period, 2 dpi (Figure 4C). The fusion events were decreased in a dose-dependent manner in the baculovirus-infected Sf21 cells. Interestingly, the fluorescence microscopy analysis and dose response curves confirmed that approximately 80 μM of UA can abrogate most cell–cell fusion events in both cases. To rule out that cell fusion was mediated through the baculovirus envelop proteins, e.g., gp64, we tested the pH effect on the baculovirus-mediated cell fusion. We found that baculoviruses vAc-EGFP can express both GP64 and EGFP, but not E1 protein of CHIKV. It cannot induce cell fusion events in the pH range of 6.6 to 5.6. We included these results in Supplementary Figure S1. Thus, vAc-CHIKV26S-Rhir-EGFP-infected insect cell should express the structural proteins of CHIKV and mediated Sf21 cell fusion. This characteristic could be used as a safe approach to screen compounds that can block the entry of CHIKV.

### 2.3. The Effects of Oleanolic Acid and Isomer Ursolic Acid on Cell Fusion Inhibition Assay

**Figure 5:**
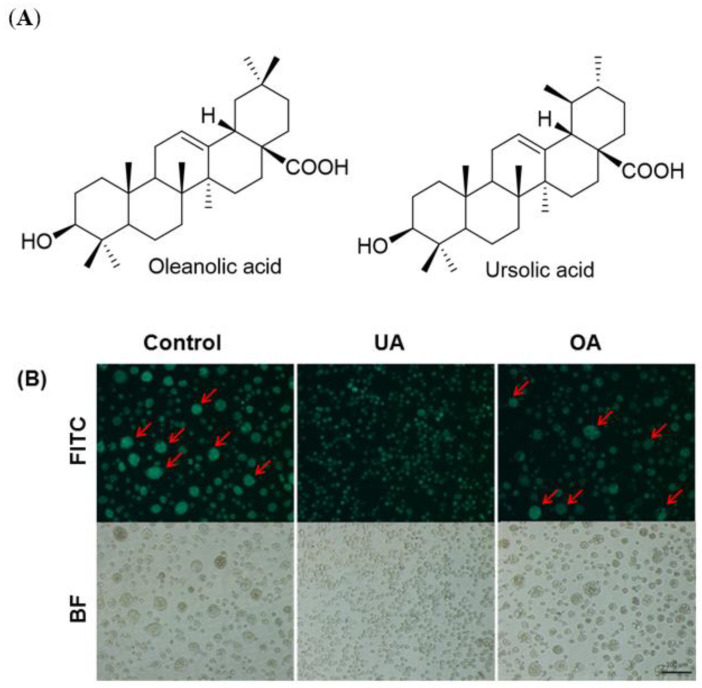
(**A**) Chemical structure of oleanolic acid (OA) and ursolic acid (UA). (**B**) Evaluation of the effects of UA and OA on CHIKV 26S-mediated Sf21 cell fusion. The Sf21 cells were infected with recombinant baculovirus vAc-CHIKV26S-Rhir-EGFP at multiplicity of infection (MOI) of 10. After 1 dpi, the infected cells were treated with 100 μM UA and OA. The arrows shows syncytial formation at 2 dpi was examined under a fluorescence microscope with a FITC channel (upper panel) and a bright field (lower panel). The bar represents 100 µm.

Ursolic acid (3β-hydroxy-urs-12-en-28-oic acid, UA) has a structural similarity tp its isomer oleanolic acid (3β-hydroxy-olea-12-en-28-oic acid, OA), shown in Figure 5A. We tried to examine the anti-fusion effects of OA in Sf21 cells infected with recombinant baculovirus at MOI 10. The cells were treated with 100 μM dosages of OA and UA. Interestingly, the fluorescence microscope studies revealed that vAc-CHIKV26S-Rhir-EGFP-infected Sf21 cells showed cell fusion in the OA-treated Sf21 cells compared with UA (Figure 5B). These results implied that BEVS-based screening approach can sensitively distinguish between the activity of UA and OA in vitro. UA is effective against CHIKV replication post-infection, as reported previously [48]. Our result suggests that cell-cell fusion was blocked by the UA and implied that the anti- CHIKV replication of UA may be through the entry block mechanism. Kuo and his team reported that Sf21 cells infected with baculovirus with CHIKV structural protein expression may be necessary for syncytium formation [35].

### 2.4. Production and Analysis of BacMam Vector System in the U-2OS Cells

**Figure 6:**
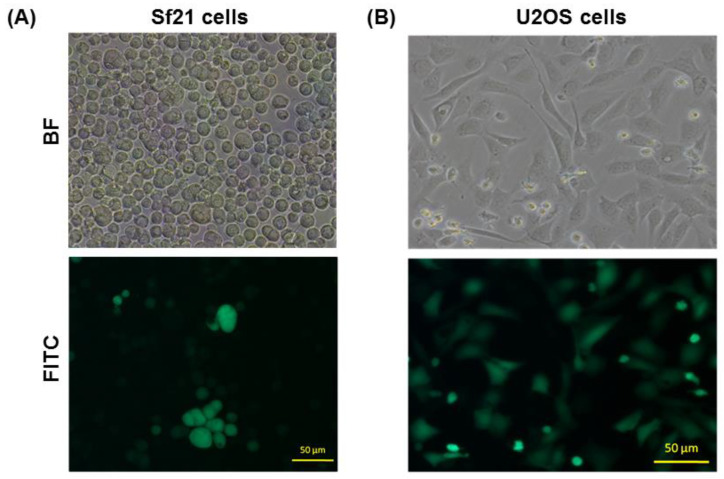
(**A**) Production and analysis of BacMam vector, vAc-CMV-CHIKV NS-EGFP in Sf21 insect cells at a multiplicity of infection (MOI) of 10 and (**B**) BacMam transduced to U-2OS cells (5 × 10^3^ cells seeded in a 24-well plate) at MOI 10. The BacMam transfection in Sf21 and transduction in U-2OS cells at 2 (dpi and dpt) were examined under a fluorescence microscope with a FITC channel (lower panel) and a bright field (upper panel) at 450/490-nm filter set using an exposure time of 1 s. The bar represents 50 µm.

To establish another strategy that can also block the replication of CHIKV in mammalian cells using BEVS, we constructed a BacMam named vAc-CMV-CHIKV NS-EGFP. vAc-CMV-CHIKV NS-EGFP is a recombinant baculovirus containing CHIKV non-structural proteins (nsP1–4) and an EGFP gene flanking the subgenomic promoter (SP) under the control of cytomegalovirus (CMV) promoter (Figure 1B). The recombinant BacMam virus, vAc-CMV-CHIKV NS-EGFP, was isolated from Sf21 cells. As shown in Figure 6A, some of the vAc-CMV-CHIKV NS-EGFP-infected Sf21 cells emitted green fluorescence which implied that the leaked transcriptional activity of the mammalian virus derived promoter CMV can support the function of replicon in Sf21 insect cells. Thus, we isolated and purified the vAc-CMV-CHIKV NS-EGFP through EGFP fluorescence under a fluorescence microscope. Then, we transduced to U-2OS cells with vAc-CMV-CHIKV NS-EGFP at a multiplicity of infection (MOI) of 10. We tested whether the transient expression of non-structural proteins with subgenomic promoter-EGFP (SP-EGFP) cassette can act as a CHIKV replicon system in U-2OS cells. As shown in Figure 6B, the vAc-CMV-CHIKV NS-EGFP-transduced U-2OS cells emitted green fluorescence and confirmed that the replicon of CHIKV could be delivered successfully to the mammalian cells through the BacMam system.

### 2.5. BacMam-Based Screening Method for Anti-CHIKV Plant Derived Compounds

**Figure 7:**
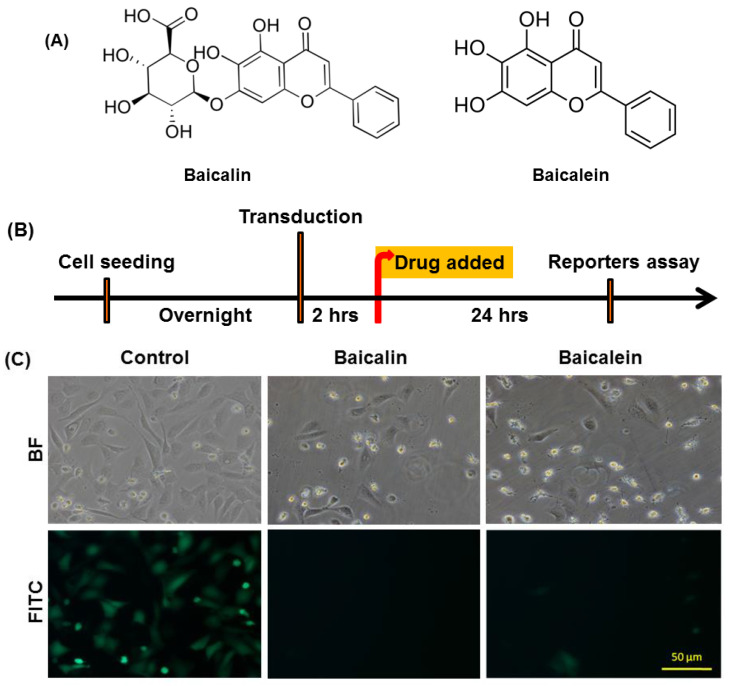
(**A**) Chemical structure of baicalin and baicalein [50]. (**B**) Schedule detailing BacMam virus transduction and plant-derived compounds treatment in U-2OS cells after 2 h of BacMam transduction. (**C**) The effects of baicalin and baicalein on BacMam-mediated replicon activity in U-2OS cells. U-2OS cells were transduced with vAc-CMV-CHIKV NS-EGFP BacMam at a multiplicity of infection (MOI) of 10. After 2 h, the transduced cells were treated with 56 nM of baicalin and 92 nM of baicalein. The replicon activity of CHIKV in mammalian cells using BacMam system at 1 dpt was examined under a fluorescence microscope with a FITC channel (lower panel) and a bright field (upper panel). The bar represents 50 µm.

Further, we intended to investigate whether BacMam-based screening method could act as a novel tool to identify anti-replicon of CHIKV activities of natural products in vitro. There is no recorded evidence for using the BacMam system to screen plant-derived anti-CHIKV compounds. Figure 7A shows the chemical structure of baicalin (5,6-dihydroxy-7-O-glucuronide flavone) and baicalein (5, 6, 7-trihydroxyflavone), which are flavonoids originally isolated from Baikal skullcap (*Scutellaria baicalensis*), a Chinese medicinal plant [49,50]. Schedule detailing vAc-CMV-CHIKV NS-EGFP BacMam transduction and screening procedures of plant-derived compounds is illustrated in Figure 7B. The BacMam transduced U-2OS cells were used to test the effects of baicalin and baicalein on CHIKV replicon activity in vitro. When the U-2OS cells were transduced with vAc-CMV-CHIKV NS-EGFP at MOI of 10 for 2 h and then treated with 56 nM of baicalin and 92 nM of baicalein, the expression of green fluorescence was reduced as observed by fluorescence microscopy (Figure 7C). This shows that baicalin and baicalein can inhibit the replicon activity of CHIKV in mammalian cells. Specifically, baicalin-treated U-2OS cells were inhibited more by the CHIKV replicon compared with baicalein treated cells.

### 2.6. Evaluation of Anti-CHIKV Ability and Dose Assessment of Baicalin through BacMam Based Drug Screening Platform

**Figure 8:**
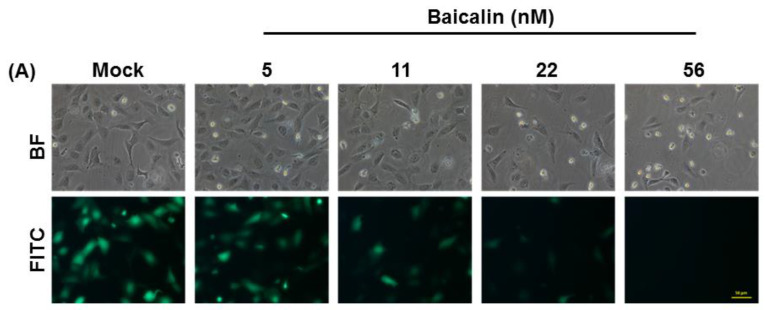

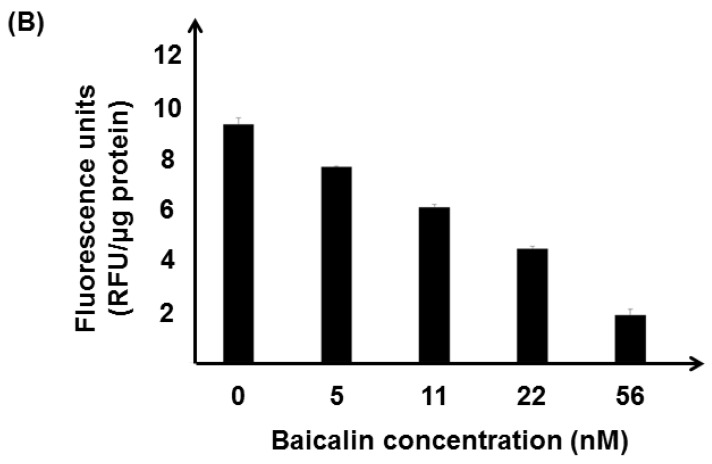
Evaluation of anti-CHIKV ability of baicalin through BacMam virus-based drug screening platform. (**A**) Effects of baicalin on vAc-CMV-CHIKV NS-EGFP BacMam mediated U-2OS cell replication at different concentrations. U-2OS cells were transduced with vAc-CMV-CHIKV NS-EGFP BacMam at a multiplicity of infection (MOI) of 10. After 2 h, the transduced cells were treated with 5, 11, 22 and 56 nM concentrations of baicalin. The replicon activity of CHIKV in mammalian cells using a BacMam system at 1 dpt was examined under a fluorescence microscope with a FITC channel (lower panel) and a bright field (upper panel). The bar represents 50 µm. (**B**) Dose response of baicalin on BacMam-mediated CHIKV replicon activity in U-2OS cells. The graph shows the anti-CHIKV effects of baicalin with respect to the control group (mock). The wavelength of emission and excitation spectra were monitored at 505 nm and 488 nm. All the data were replicated 3 times.

Baicalin and baicalein can inhibit BacMam-mediated CHIKV replicon activity; therefore, both compounds might be able to suppress CHIKV infection. To confirm the dose level of the anti-CHIKV effects of baicalin against intracellular CHIKV replicon activity in mammalian cells, the vAc-CMV-CHIKV NS-EGFP BacMam transduced to U-2OS cells were treated with varied concentrations of baicalin (5, 11, 22 and 56 nM) at 37 °C for a 24-h incubation period. The inoculum was then removed and plant-derived compound-treated cells were subjected to fluorescence analysis to determine the effect of baicalin on CHIKV replicon activity in a dose dependent manner (Figure 8A). Our investigations showed that baicalin at a concentration of 56 nM completely inhibited CHIKV replicon activity in the BacMam-transduced mammalian cells. The lower level of baicalin treatment showed that, in most of the U-2OS cells, CHIKV replicons were occurring in vitro (up to a 22-nM concentration from the images). Furthermore, Figure 8B confirmed that the quantity of the fluorescent protein expressed after treatment with baicalin is in a dose-dependent manner. The highest EGFP protein expression was observed in the baicalin untreated U-2OS cells and that the level gradually decreased as the concentration of treated compounds increased. We also used MTT assay to analyze the cell viability of U-2OS cells transduced with vAc-CMV-CHIKV NS-EGFP BacMam treated with different concentrations of baicalin and baicalein. As shown in Supplementary Figure S2, baicalin and baicalein did not show significant toxicity below 200 nM. Baicalein even enhanced cell viability at concentrations less than 300 nM.

Both baicalin and baicalein showed their inhibition of CHIKV replicon activity in U-2OS cells. Interestingly, the protein quantification results and the dose response curves confirmed the anti-CHIKV effect of baicalin more explicitly. This study confirmed that the BacMam systems can be a tool for screening plant-derived products against CHIKV infection through replicon inhibition.

**Figure 9:**
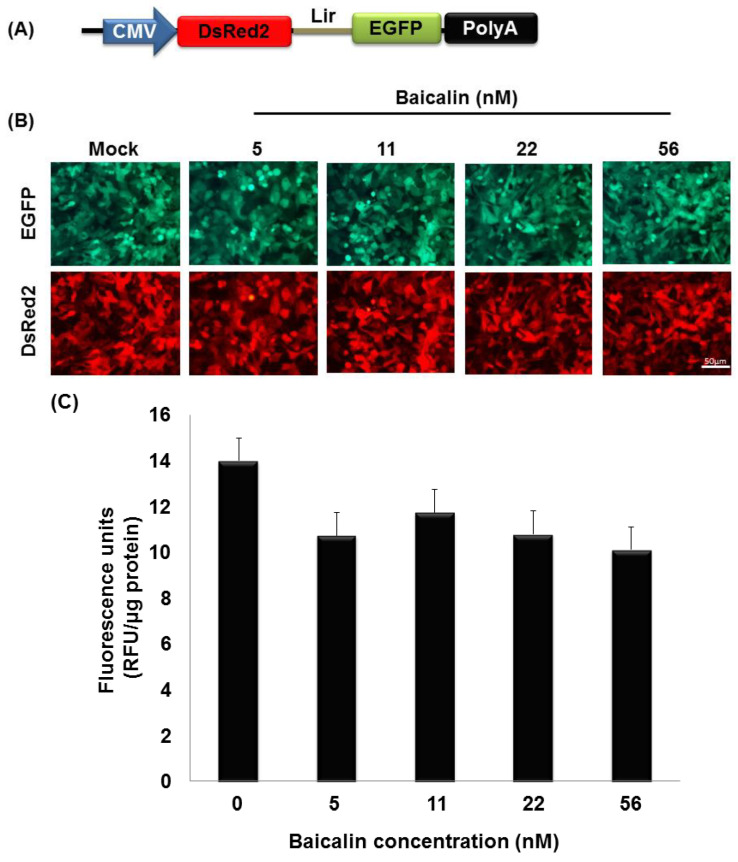

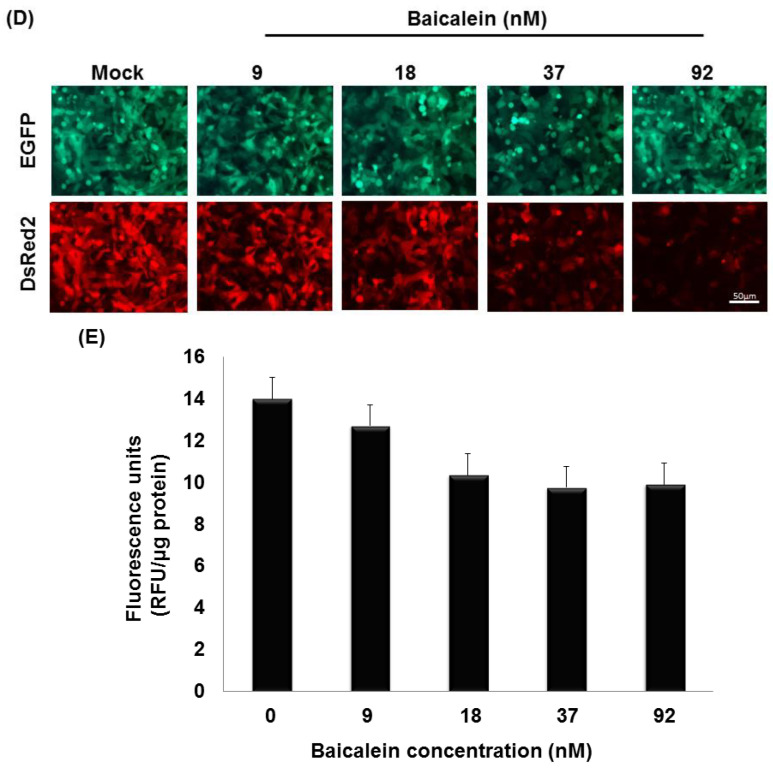
Schematic presentation of the BacMam vector with a CMV promoter and its effects on plant-derived compounds. (**A**) vAc-CMV-DsRed2-Lir-EGFP BacMam vector contain CMV to control the expression of the reporter genes DsRed2 and the EGFP flanking with Lir. DsRed2, Red fluorescent protein; Lir, a chimeric IRES; and EGFP, enhanced green fluorescent protein gene. (**B**) Effects of baicalin on vAc-CMV-DsRed2-Lir-EGFP BacMam-mediated reporter genes expression in U-2OS cell at different concentrations. The bar represents 50 µm. (**C**) DeRed fluorescent quantitation of different concentrations of baicalin on BacMam-mediated CMV promoter activity in U-2OS cells. (**D**) Effects of baicalein on vAc-CMV-DsRed2-Lir-EGFP BacMam-mediated reporter genes expression in U-2OS cell at different concentrations. (**E**) DeRed fluorescent quantitation of different concentrations of baicalein on BacMam-mediated CMV promoter activity in U-2OS cells. For (**C**,**E**) the wavelength of emission was monitored at 583 nm and excitation stimulated at 558 nm. All data were replicated 3 times.

Further, we intended to rule out the observation that baicalin and baicalein can inhibit BacMam-mediated CHIKV replicon activity, shown in Figure 8, through the interruption of the transcriptional activity of the CMV promoter. vAc-CMV-DsRed2-Lir-EGFP BacMam containing red fluorescent protein and EGFP gene flanking the Lir, a chimeric IRES under the control of the CMV promoter vector system was constructed, as shown in Figure 9A. vAc-CMV-DsRed2-Lir-EGFP BacMam can express red fluorescence, as well as EGFP, in the transduced U-2OS cells, as shown in Figure 9B. Thus, this vector was used to check whether the transcriptional activity of the CMV promoter is affected by baicalin and baicalein in the BacMam-transduced U-2OS cells. Schedule detail of vAc-CMV-DsRed2-Lir-EGFP BacMam transduction and the screening procedure of plant-derived compounds is followed, as depicted in Figure 7B. However, as we transduced U-2OS with vAc-CMV-DsRed2-Lir-EGFP at MOI of 10, the transduction rate was low (data not shown). This implies that the replicon of CHIKV does “amplify” EGFP expression under the control of the SP promoter in vAc-CMV-CHIKV NS-EGFP BacMam-transduced U-2OS cells at a MOI of 10, as shown in Figure 7A. Thus, the U-2OS cells were transduced with vAc-CMV-DsRed2-Lir-EGFP at a MOI of 100 for 2 h and then separately treated with various concentrations of baicalin (5, 11, 22 and 56 nM) and baicalein (9, 18, 37 and 92 nM) at 37 °C for a 24-h incubation period. The inoculum was then removed and the cells were subjected to fluorescence analysis to determine the effect of baicalin and baicalein on the CMV promoter in a dose-dependent manner (Figure 9B–E). Our results showed that there is no obvious effect of baicalin and baicalein on CMV promoter as the red and green fluorescence expressions were not significantly altered under fluorescence microcopy (Figure 9B,D). In addition, the quantitation of DsRed protein expression was also done through a spectrofluorometer (Figure 9C,E). Altogether, the results indicated that baicalin and baicalein had only a minimal effect on the CMV promoter without any dose-dependent correlation. Thus, we confirmed that baicalin and baicalein can inhibit BacMam-mediated CHIKV replicon activity and not by the CMV promoter.

## 3. Discussion

Chikungunya fever is a vector-borne viral disease transmitted to humans by CHIKV-infected mosquitoes. There have been many outbreaks of CHIKV infection worldwide, and the virus poses ongoing risks to global health [51]. To date, there is no licensed vaccine or effective antiviral therapy for prevention and treatment of Chikungunya infection. Therefore, drug discovery and screening natural products or plant-derived compounds against CHIKV remains a priority. CHIKV is classified as a risk group-3 pathogen; however, the baculovirus- and BacMam-based expression systems are safe and can be operated in a BSL-1 laboratory [34]. The baculovirus bi-cictronic expression system (BEVS) was used to demonstrate a flexible drug screening platform for CHIKV. The BEVS was used to co-express both EGFP and CHIKV structural protein in Sf21 cells and the induction of Sf21 cell fusion. Therefore, using vAc-CHIKV26S-Rhir-EGFP-mediated fusion inhibition assay in Sf21 cells can decrease the risk of a drug screening platform to search for novel anti-CHIKV compounds. From this study, ursolic acid (UA) shows the ability to block cell fusion events mediated by CHIKV structural proteins and confirmed anti-CHIKV effects (Figure 4 and Figure 5). UA showed cell fusion inhibition abilities after treatment with a concentration of 80 μM in insect cells. CHIKV has two envelope glycoproteins, E1 and E2, which cover the viral surface with spike structures and mediate viral entry into host cells [52,53]. The structural proteins, E2 are responsible for cell attachment, while E1 controls membrane fusion during viral infection [35,54,55]. It has already been reported that the successful induction of cell fusion and syncytium formation in Sf21 cells infected with the recombinant baculovirus expressed only 6K-E1 proteins [35]. Interestingly, the UA structural isomer oleanolic acid (OA) was found to be less active than UA against CHIKV infection and cannot inhibit cell fusion events in Sf21 cells (Figure 5B). The naturally occurring pentacyclic triterpenoids are UA and OA containing both hydroxyl groups and carboxylic groups (Figure 5A), however previous reports have suggested that the activity of these compounds are related to their basic triterpenoid skeletal structure and the attached functional groups offer opportunities for chemical modifications. Ursolic acid is a plant-derived pentacyclic triterpenoid acid that shows anti-CHIKV effects and inhibits CHIKV infection in vitro [56]. Both UA and OA can improve their biological activities due to chemical structural changes [57,58,59]. Previous studies have indicated that OA was less active than UA in inhibition of antiviral activities in human enterovirus 71 and H5N1 infection [60,61]. Based on these findings, we suggest that UA might possess potent anti-CHIKV abilities compared to OA in suppressing CHIKV infection and can be used as a drug against Chikungunya fever. Our results showed that the BacMam-based anti-CHIKV drug screening platform has broad applicability in future research. The experiments confirmed newly generated replicon BacMam expressing CHIKV non-structural proteins with enhanced green fluorescent protein (SP-EGFP) in Sf21 and mammalian cells (Figure 6A). The recombinant baculoviruses engineered to contain a mammalian expression cassette have been shown to efficiently transduce to U-2OS cells. This BacMam system has evolved rapidly over the last few years for the purpose of drug screening and protein structural studies [32,62,63,64]. Despite significant gaps in the clinical literature and previous studies, anti-CHIKV effects of plant derived compounds were found; however, synergistic anti-CHIKV effects of baicalin in silico have been reported [65]. The baicalin and baicalein flavonoids have the potential to inhibit CHIKV infection in mammalian cells [66,67], although the details of the mechanisms are not known. In this study, baicalin and baicalein were originally isolated from plant sources and showed the ability to block BacMam-mediated replicon activity in U-2OS cells (Figure 6). These results were consistent with a previous study in which baicalin was a potential inhibitor of CHIKV using a computational approach. The study showed that baicalin interacted with non-structural protein 3 (nsP3) of CHIKV as one of the most important viral elements in CHIKV intracellular replication [65]. Furthermore, we also demonstrated the baicalin and baicalein did not affect the transcriptional activity of the CMV promoter (Figure 9). It will be interesting to know whether baicalin or baicalein interfere in the processes of nsP1-nsP4 polyproteins or the assembly of functional replicase. In the future, we will try to explore the detailed mechanisms of baicalin and baicalein through studies of these compounds on (1) the process of and assembly of nsp1-nsP4, and (2) the RNA level of the reporter EGFP gene. We will investigate whether baicalin and baicalein interact with the nsP3 of CHIKV directly, in the future. Additionally, Q-PCR will be employed to dissect the action of baicalin and baicalein on the replicon of CHIKV at the molecular level. In conclusion, our results demonstrate that the baculovirus-based protein expression system, BEVS, and the gene delivery system, BacMam, could work together to act an anti-CHIKV drug screening platform.

## 4. Materials and Methods

### 4.1. Cells, Recombinant Baculoviruses and Transfection

The *Spodoptera frugiperda* IPBL-Sf21 (Sf21) cell line was cultured in TNM-FH insect medium containing 8% heat-inactivated fetal bovine serum (FBS) at 27 °C [68]. The U-2OS (human osteogenic sarcoma cell line, purchased from Bioresource Collection and Research Center 331, Shih-Pin Rd., Hsinchu 300193, Taiwan) cells were grown in McCoy’s medium supplemented with 10% fetal bovine serum [69]. Cellfectin (Invitrogen, Carlsbad, CA, USA) was used for transfection in Sf21 cells according to the manufacturer’s protocol. Recombinant viruses were collected from Sf21 cell cultures emitting green fluorescence under a fluorescence microscope (Nikon, Tokyo, Japan). Sf21 monolayers were used for virus propagation and determined the viral titers according to standard protocols described by O’Reilly and Miller et al. [70].

### 4.2. Transduction of Mammalian Cells

The U-2OS cells were seeded in 24-well plates at 5 × 10^3^ cells/well. The culture medium was removed and replaced with virus inoculate at a multiplicity of infection (MOI) of 10, and centrifuged at 600× *g* for 1 h. Then, the supernatant was removed, and a fresh medium containing 5 mM sodium butyrate was added and cultured at 37 °C [70]. The varied concentrations of plant-derived compounds (Table S1) were added after 2 h of transduction. The replicon activity of CHIKV in U-2OS cells was examined under a fluorescence microscope after 1 dpt.

### 4.3. Construction of Transfer Vectors

DNA preparations and manipulations were performed using standard methods as described by Sambrook and co-workers [71], or protocol provided by the manufacturers of the reagents. An established bi-cistronic recombinant baculovirus used in this study: vAc-CHIKV26S-Rhir-EGFP (full length gene expressing both E1 and E2 proteins with green fluorescence) and BacMam vector vAc-CMV-CHIKV NS-EGFP (full length gene expressing both non-structural proteins with green fluorescence) were prepared as described in [19,35]. All construct sequences were confirmed by DNA sequencing.

### 4.4. Fusion Inhibition Assay

In previous studies, we had demonstrated that insect Sf21 cells infected with the recombinant baculoviruses expressed structural proteins of CHIKV and would induce membrane fusion [35] and indicated this fusion event through the E1 protein of CHIKV [19]. Briefly, Sf21 cells were infected with vAc-CHIKV26S-Rhir-EGFP at an MOI of 1. After one dpi, the culture medium was replaced with Sf-900 II SFM containing 2% FCS and cholesterol (100 μg/mL). The cellular fusion event was observed and photographed using an inverted fluorescence microscope (Nikon, Tokyo, Japan) and we established the methods as described in the protocol [34]. The average size of a single syncytial formation was determined by counting at least three nuclei in each cell–cell fusion.

### 4.5. Spectrofluorometer Quantification

We followed the same quantification procedure for fluorescence measurement from our previous studies [72]. For EGFP measurements, cells transduced with vAc-CMV-CHIKV NS-EGFP were washed with phosphate-buffered saline (PBS) and lysed with 50 µL RIPA buffer (150 mM NaCl, 1% Triton X-100, 0.5% sodium deoxycholate, 0.1% SDS, 50 mM Tris pH 8.0). A 30-µL extract was used for EGFP measurement and a 5-µL extract was used for protein quantity. The green fluorescence intensities were measured using a Cary Eclipse Fluorescence spectrophotometer (Agilent Technologies, Santa Clara, CA, USA). The protein quantities were measured using a BCA protein assay (Thermo Fisher Scientific, Waltham, MA, USA) with BSA as a standard, and was used to normalize the green fluorescence intensities. For this purpose, the green fluorescence intensities were normalized with total protein and expressed as fluorescence units per µg protein (FU/µg protein).

## 5. Conclusions

In this article, we report the application of a novel BEVS and BacMam system for the screening anti-CHIKV plant-derived compounds. This screening system has potential application for the development of novel drugs against Chikungunya fever and other alphavirus infections. This study presents the materials for insect culture and preparations of recombinant baculovirus through a bi-cistronic baculovirus transfer vector. We explained the procedures employing expressed CHIKV non-structural proteins in mammalian cells through a BacMam system to screen for candidate compounds that prevent CHIKV replication and identified the activity between isomers in vitro. In addition, we also we show the methods of an in vitro anti-CHIKV activity assay to validate compounds identified in the recombinant baculovirus-mediated and BacMam mediated system. Altering formulations of chemicals or combinations with other anti-CHIKV drugs were able to enhance drug efficacy and reduce their dosage range; in addition, through structure modification to find some new derivatives, we also a different way to produce efficacious and safe anti-CHIKV drugs.

## Supplementary Materials

The following are available online at https://www.mdpi.com/article/10.3390/ijms22157891/s1.
