# Supplementary material for: Recombinant Baculovirus: A Flexible Drug Screening Platform for Chikungunya Virus
Source: Int J Mol Sci. 2021 Jul 23;22(15):7891. doi: 10.3390/ijms22157891 (PMC8347121; doi:10.3390/ijms22157891)
Supplement: Supplementary file 1 [file ijms-22-07891-s001.zip › ijms-1291024-supplementary.pdf]

Table S1. Natural compounds selected for the screening of anti-CHIKV drugs through BEVS.

Compound name	Fusion inhibitor	Replicon inhibitor	Known effects & functions	Reference
Amygdalin	No	No	Reduces lipopolysaccharide-induced chronic liver injury	[1]
Baicalein	No	Yes	Anti-diabetic, anti-cancer activity	[2, 3]
Baicalin	No	Yes	Anti-bacterial, anti-inflammatory activity	[4, 5]
Brazilein	No	No	Anti-cancer, anti-inflammatory activity	[6, 7]
Lucidone	No	No	Anti-inflammatory, anti-viral activity	[8, 9]
Mangiferin	No	No	Anti-gestational diabetes mellitus, suppresses placental oxidative stress and inflammation	[10, 11]
Melatonin	No	No	Anti-diabetic, anti-cancer activity	[12, 13]
Orlistat	No	No	Anti-cancer activity	[14]
Phycocyanin	No	No	Anti-proliferative effects in cancer	[15]
Rb1	Yes	No	Induce diabetics and improve cardiac dysfunction	[16]
Rg1	No	No	Anti-diabetic, anti-cancer and anti-inflammatory effects	[17-19]
Rg3	No	No	Anti-cancer effects	[20]
Rh3	No	No	Anti-inflammatory mechanism	[21]
Rosmarinic acid	No	No	Anti-cancer, anti-viral and anti-inflammatory effects	[22-24]
Sofalcone	No	No	Anti-microbial activity	[25]
Sulforaphane	No	No	Anti-cancer activity	[26]
Ursolic acid	Yes	No	Anti-cancer, anti-diabetic and anti-viral properties	[27-29]

Figure S1.

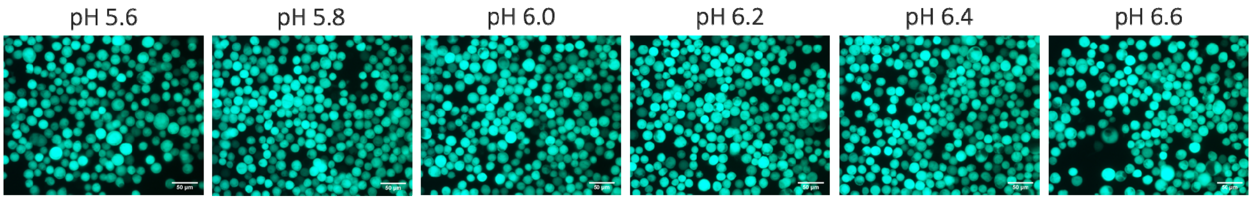

The pH titration to test the baculovirus mediated cell fusion. The baculoviruses vAc-EGFP can express both GP64 and EGFP, but not E1 protein of CHIKV. It cannot induce cell fusion events in the pH range of 6.6 to 5.6.

Figure S2: Cell viability assay

(A)

(B)

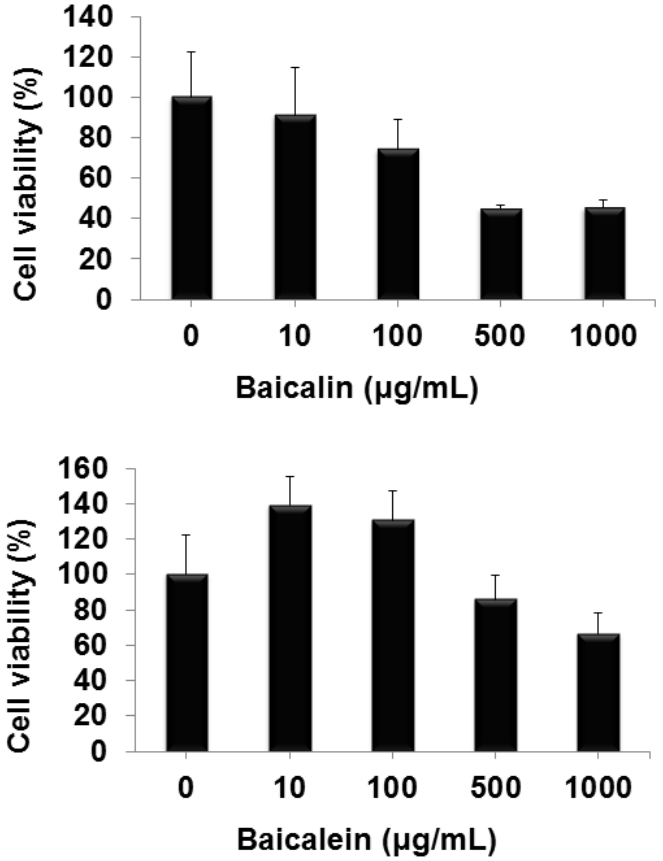

Figure S2: MTT assay analysis of cell viability of U-2OS cells treated by different concentrations of baicalin and baicalein. The cells were transduced with vAc-CMV-CHIKV NS-EGFP BacMam at multiplicity of infection (MOI) of 10. After 2 hrs, the transduced cells were treated with 10, 100, 500 and 1000 ug/mL plant derived compounds. After incubation for 24 hrs, cell viability was determined.

Reference

1. Yang, Y.; Zhao, J.; Song, X.; Li, L.; Li, F.; Shang, J.; Wang, W. Amygdalin reduces lipopolysaccharide-induced chronic liver injury in rats by down-regulating PI3K/AKT, JAK2/STAT3 and NF-κB signalling pathways. *Artif. Cells, Nanomedicine, Biotechnol.* **2019**, *47*, 2688–2697, doi:10.1080/21691401.2019.1634084.
2. Dou, J.; Wang, Z.; Ma, L.; Peng, B.; Mao, K.; Li, C.; Su, M.; Zhou, C.; Peng, G. Baicalein and baicalin inhibit colon cancer using two distinct fashions of apoptosis and senescence. *Oncotarget* **2018**, *9*, 20089–20102, doi:10.18632/oncotarget.24015.
3. Zhang, B.; Sun, W.; Yu, N.; Sun, J.; Yu, X.; Li, X.; Xing, Y.; Yan, D.; Ding, Q.; Xiu, Z.; et al. Anti-diabetic effect of baicalein is associated with the modulation of gut microbiota in streptozotocin and high-fat-diet induced diabetic rats. *J. Funct. Foods* **2018**, *46*, 256–267, doi:10.1016/j.jff.2018.04.070.
4. Peng, L.-Y.; Yuan, M.; Wu, Z.-M.; Song, K.; Zhang, C.-L.; An, Q.; Xia, F.; Yu, J.-L.; Yi, P.-F.; Fu, B.-D.; et al. Anti-bacterial activity of baicalin against APEC through inhibition of quorum sensing and inflammatory responses. *Sci. Rep.* **2019**, *9*, 4063, doi:10.1038/s41598-019-40684-6.
5. Shen, K.; Feng, X.; Pan, H.; Zhang, F.; Xie, H.; Zheng, S. Baicalin Ameliorates Experimental Liver Cholestasis in Mice by Modulation of Oxidative Stress, Inflammation, and NRF2 Transcription Factor. *Oxidative Med. Cell. Longev.* **2017**, *2017*, 1–11, doi:10.1155/2017/6169128.
6. Mou, Z.; Wang, Y.; Li, Y. Brazilein induces apoptosis and G1/G0 phase cell cycle arrest by up-regulation of miR-133a in human vestibular schwannoma cells. *Exp. Mol. Pathol.* **2019**, *107*, 95–101, doi:10.1016/j.yexmp.2018.12.010.
7. Yan, X.-J.; Chai, Y.-S.; Yuan, Z.-Y.; Wang, X.-P.; Jiang, J.-F.; Lei, F.; Xing, D.-M.; Du, L.-J. Brazilein inhibits neuronal inflammation induced by cerebral ischemia and oxygen-glucose deprivation through targeting NOD2 expression. *Chin. J. Nat. Med.* **2016**, *14*, 354–62, doi: 10.3724/SP.J.1009.2016.00354.
8. Feng, X.; Wang, Y. Anti-inflammatory, anti-nociceptive and sedative-hypnotic activities of lucidone D extracted from Ganoderma lucidum. *Cell. Mol. Biol.* **2019**, *65*, 37–42, doi:10.14715/cmb/2019.65.4.6.
9. Chen, W.-C.; Tseng, C.-K.; Lin, C.-K.; Wang, S.-N.; Wang, W.-H.; Hsu, S.-H.; Wu, Y.-H.; Hung, L.-C.; Chen, Y.-H.; Lee, J.-C. Lucidone suppresses dengue viral replication through the induction of heme oxygenase-1. *Virulence* **2018**, *9*, 588–603, doi:10.1080/21505594.2017.1421893.
10. Sha, H.; Zeng, H.; Zhao, J.; Jin, H. Mangiferin ameliorates gestational diabetes mellitus-induced placental oxidative stress, inflammation and endoplasmic reticulum stress and improves fetal outcomes in mice. *Eur. J. Pharmacol.* **2019**, *859*, 172522, doi:10.1016/j.ejphar.2019.172522.
11. Huang, J.; Zheng, L.; Wang, F.; Su, Y.; Kong, H.; Xin, H. Mangiferin ameliorates placental oxidative stress and activates PI3K/Akt/mTOR pathway in mouse model of preeclampsia. *Arch. Pharmacal Res.* **2020**, *43*, 233–241, doi:10.1007/s12272-020-01220-7.
12. Ebaid, H.; Bashandy, S.A.E.; Abdel-Mageed, A.M.; Al-Tamimi, J.; Hassan, I.; Alhazza, I.M. Folic acid and melatonin mitigate diabetic nephropathy in rats via inhibition of oxidative stress. *Nutr. Metab.* **2020**, *17*, 1–14, doi:10.1186/s12986-019-0419-7.
13. Reiter, R.J.; Rosales-Corral, S.A.; Tan, D.-X.; Acuna-Castroviejo, D.; Qin, L.; Yang, S.-F.; Xu, K. Melatonin, a Full Service Anti-Cancer Agent: Inhibition of Initiation, Progression and Metastasis. *Int. J. Mol. Sci.* **2017**, *18*, 843, doi:10.3390/ijms18040843.
14. Czumaj, A.; Zabielska, J.; Pakiet, A.; Mika, A.; Rostkowska, O.; Makarewicz, W.; Kobiela, J.; Sledzinski, T.; Stelmanska, E. In Vivo Effectiveness of Orlistat in the Suppression of Human Colorectal Cancer Cell Proliferation. *Anticancer. Res.* **2019**, *39*, 3815–3822, doi:10.21873/anticanres.13531.
15. Hao, S.; Li, S.; Wang, J.; Yan, Y.; Ai, X.; Zhang, J.; Ren, Y.; Wu, T.; Liu, L.; Wang, C. Phycocyanin Exerts Anti-Proliferative Effects through Down-Regulating TIRAP/NF-κB Activity in Human Non-Small Cell Lung Cancer Cells. *Cells* **2019**, *8*, 588, doi:10.3390/cells8060588.
16. Qin, L.; Wang, J.; Zhao, R.; Zhang, X.; Mei, Y. Ginsenoside-Rb1 Improved Diabetic Cardiomyopathy through Regulating Calcium Signaling by Alleviating Protein O-GlcNAcylation. *J. Agric. Food Chem.* **2019**, *67*, 14074–14085, doi:10.1021/acs.jafc.9b05706.
17. Gao, Y.; Li, J.; Chu, S.; Zhang, Z.; Chen, N.; Li, L.; Zhang, L. Ginsenoside Rg1 protects mice against streptozotocin-induced type 1 diabetic by modulating the NLRP3 and Keap1/Nrf2/HO-1 pathways. *Eur. J. Pharmacol.* **2020**, *866*, 172801, doi:10.1016/j.ejphar.2019.172801.

18. Tao, T.; Chen, F.; Bo, L.; Xie, Q.; Yi, W.; Zou, Y.; Hu, B.; Li, J.; Deng, X. Ginsenoside Rg1 protects mouse liver against ischemia–reperfusion injury through anti-inflammatory and anti-apoptosis properties. *J. Surg. Res.* **2014**, *191*, 231–238, doi:10.1016/j.jss.2014.03.067.
19. Chu, Y.; Zhang, W.; Kanimozhi, G.; Brindha, G.R.; Tian, D. Ginsenoside Rg1 Induces Apoptotic Cell Death in Triple-Negative Breast Cancer Cell Lines and Prevents Carcinogen-Induced Breast Tumorigenesis in Sprague Dawley Rats. *Evidence-Based Complement. Altern. Med.* **2020**, *2020*, 1–12, doi:10.1155/2020/8886955.
20. Liang, Y.; Zhang, T.; Jing, S.; Zuo, P.; Li, T.; Wang, Y.; Xing, S.; Zhang, J.; Wei, Z. 20(S)-Ginsenoside Rg3 Inhibits Lung Cancer Cell Proliferation by Targeting EGFR-Mediated Ras/Raf/MEK/ERK Pathway. *Am. J. Chin. Med.* **2021**, *49*, 753–765, doi:10.1142/s0192415x2150035x.
21. Lee, Y.Y.; Park, J.-S.; Lee, E.-J.; Lee, S.-Y.; Kim, D.-H.; Kang, J.L.; Kim, H.-S. Anti-inflammatory Mechanism of Ginseng Saponin Metabolite Rh3 in Lipopolysaccharide-Stimulated Microglia: Critical Role of 5′-Adenosine Monophosphate-Activated Protein Kinase Signaling Pathway. *J. Agric. Food Chem.* **2015**, *63*, 3472–3480, doi:10.1021/jf506110y.
22. Luo, C.; Zou, L.; Sun, H.; Peng, J.; Gao, C.; Bao, L.; Ji, R.; Jin, Y.; Sun, S. A Review of the Anti-Inflammatory Effects of Rosmarinic Acid on Inflammatory Diseases. *Front. Pharmacol.* **2020**, *11*, 153, doi:10.3389/fphar.2020.00153.
23. Anwar, S.; Shamsi, A.; Shahbaaz, M.; Queen, A.; Khan, P.; Hasan, G.M.; Islam, A.; Alajmi, M.F.; Hussain, A.; Ahmad, F.; et al. Rosmarinic Acid Exhibits Anticancer Effects via MARK4 Inhibition. *Sci. Rep.* **2020**, *10*, 1–13, doi:10.1038/s41598-020-65648-z.
24. Hsieh, C.-F.; Jheng, J.-R.; Lin, G.-H.; Chen, Y.-L.; Ho, J.-Y.; Liu, C.-J.; Hsu, K.-Y.; Chen, Y.-S.; Chan, Y.F.; Yu, H.-M.; et al. Rosmarinic acid exhibits broad anti-enterovirus A71 activity by inhibiting the interaction between the five-fold axis of capsid VP1 and cognate sulfated receptors. *Emerg. Microbes Infect.* **2020**, *9*, 1194–1205, doi:10.1080/22221751.2020.1767512.
25. Dan, W.; Dai, J. Recent developments of chalcones as potential antibacterial agents in medicinal chemistry. *Eur. J. Med. Chem.* **2020**, *187*, 111980, doi:10.1016/j.ejmech.2019.111980.
26. Su, X.; Jiang, X.; Meng, L.; Dong, X.; Shen, Y.; Xin, Y. Anticancer Activity of Sulforaphane: The Epigenetic Mechanisms and the Nrf2 Signaling Pathway. *Oxidative Med. Cell. Longev.* **2018**, *2018*, 1–10, doi:10.1155/2018/5438179.
27. Hussain, H.; Green, I.R.; Ali, I.; Khan, I.A.; Ali, Z.; Al-Sadi, A.; Ahmed, I. Ursolic acid derivatives for pharmaceutical use: a patent review (2012-2016). *Expert Opin. Ther. Patents* **2017**, *27*, 1061–1072, doi:10.1080/13543776.2017.1344219.
28. Guzmán-Ávila, R.; Flores-Morales, V.; Paoli, P.; Camici, G.; Ramirez-Espinosa, J.J.; Cerón-Romero, L.; Navarrete-Vázquez, G.; Hidalgo-Figueroa, S.; Yolanda Rios, M.Y.; Villalobos-Molina, R.; et al. Ursolic acid derivatives as potential antidiabetic agents: In vitro , in vivo , and in silico studies. *Drug Dev. Res.* **2018**, *79*, 70–80, doi:10.1002/ddr.21422.
29. Tohmé, M.J.; Giménez, M.; Peralta, A.; Colombo, M.; Delgui, L. Ursolic acid: A novel antiviral compound inhibiting rotavirus infection in vitro. *Int. J. Antimicrob. Agents* **2019**, *54*, 601–609, doi:10.1016/j.ijantimicag.2019.07.015.

.
